# Membrane Repairing Capability of Non-Small Cell Lung Cancer Cells Is Regulated by Drug Resistance and Epithelial-Mesenchymal-Transition

**DOI:** 10.3390/membranes12040428

**Published:** 2022-04-15

**Authors:** Xingyu Xia, Hanbo Yang, Dennis Wai-Yin Au, Syrus Pak-Hei Lai, Yuan Lin, William Chi-Shing Cho

**Affiliations:** 1 Department of Mechanical Engineering, The University of Hong Kong, Hong Kong, China; u3005822@connect.hku.hk (X.X.); u3008009@connect.hku.hk (H.Y.); 2 Department of Clinical Oncology, Queen Elizabeth Hospital, Hong Kong, China; awy931@ha.org.hk; 3 Department of Clinical Oncology, The University of Hong Kong, Hong Kong, China; syruslai@connect.hku.hk; 4 Advanced Biomedical Instrumentation Centre, Hong Kong Science Park, Shatin, New Territories, Hong Kong, China

**Keywords:** cell membrane, atomic force microscope (AFM), membrane recovery, annexin, epithelial-mesenchymal-transition (EMT), non-small cell lung cancer, drug resistance

## Abstract

The plasma membrane separates the interior of the cells from the extracellular fluid and protects the cell from disruptive external factors. Therefore, the self-repairing capability of the membrane is crucial for cells to maintain homeostasis and survive in a hostile environment. Here, we found that micron-sized membrane pores induced by cylindrical atomic force microscope probe puncture resealed significantly (~1.3–1.5 times) faster in drug-resistant non-small cell lung cancer (NSCLC) cell lines than in their drug-sensitive counterparts. Interestingly, we found that such enhanced membrane repairing ability was due to the overexpression of annexin in drug-resistant NSCLC cells. In addition, a further ~50% reduction in membrane resealing time (i.e., from ~23 s to ~13 s) was observed through the epithelial-mesenchymal-transition, highlighting the superior viability and potential of highly aggressive tumor cells using membrane resealing as an indicator for assessing the drug-resistivity and pathological state of cancer.

## 1. Introduction

In addition to serving as the physical barrier separating cytoplasm from outside, and protecting the cell from disruptive extracellular factors, the plasma membrane also allows a cell to achieve signal and mass exchange with the surrounding microenvironment (through various transmembrane signaling proteins and ion channels/pumps) and maintain homeostasis is critical for the cell’s survival. For example, it is widely reported that cells must actively pump out calcium ions to keep their internal concentration low, a condition that is critical for the proper functioning of many proteins and signaling pathways [1]. However, damage to the membrane will enable calcium to flow into the cell, disrupting its normal function and eventually leading to proteolysis and cell death [2]. Given that membrane injuries have been commonly observed in migrating cancer cells [3], the self-repairing capability of plasma membrane will be crucial for their survival in the hostile environment [4].

Microscopically, it is well-documented that the calcium influx through the ruptured membrane could trigger a cascade of events to help its repairing. In particular, annexin family proteins (known to facilitate membrane repair by inducing proper membrane morphology changes as well as generating forces for bringing ruptured membrane together [5,6,7,8,9] will be recruited to the damage site through the binding between their negatively charged phospholipids and calcium ion [10]. On the other hand, it has also been shown that diffusion of lipids itself is enough to lead to spontaneous resealing of the membrane hole when its size is below a threshold value [11].

Interestingly, a recent study showed that membrane resealing in tumor cells is much faster than the corresponding normal ones [12]. Motivated by this exciting finding, we report a study focusing on how the membrane resealing response of non-small-cell lung cancer (NSCLC) cells is influenced by their drug-resistivity and pathological state. We chose NSCLC here because it accounts for ~85% of all lung cancers with most deaths. For this reason, intense efforts have been devoted to developing new strategies/methods for the monitoring and treatment of this disease. In particular, epidermal growth factor receptor (EGFR) was identified as an important drug target due to its critical role in signaling pathways, for example, Ras-Raf-MEK-ERK-MAPK, phosphatidylinositol 3-kinase (PI3K)/Akt, phospholipase Cγ (PLCγ) and Src kinase pathways [13,14]. Indeed, as one of the most commonly used drugs to treat NSCLC patients with mutant EGFR, erlotinib is a commonly used first-line EGFR-targeting tyrosine kinase inhibitor (TKI) [15]. In general, the mechanism for TKIs to work is to bind to specific receptors like EGFR and ALK, in competition with other ligands such as ATP, block the docking site for downstream effectors in the tyrosine kinase domain of these receptors and eventually prevent the activation of the receptor. As a result, the anti-apoptotic pathway and other uncontrolled downstream cascades eliciting tumor cell proliferation and angiogenesis will be inhibited. Unfortunately, after repeated treatments, NSCLC cells may develop resistance to erlotinib and consequently greatly reduce its efficacy [16,17]. Another common way for NSCLC cells to acquire EGFR-TKI resistance is the epithelial-mesenchymal transition (EMT) where cells undergo a switch from being epithelial- to mesenchymal-like. In this study, protocols for developing the resistance to erlotinib and triggering EMT in different NSCLC cell lines were established. We then systematically examined how these changes affect the membrane repairing capability of NSCLC cells as well as the mechanisms behind them.

## 2. Materials and Methods

### 2.1. Cell Culture

Two NSCLC cell lines, HCC4006 and ETCC016 (from Caucasian and Chinese patients, respectively) were used in this study. The HCC4006 cell line was cultured in RPMI 1640 medium with 10% fetal bovine serum (FBS) and 1% penicillin-streptomycin (PS), and ETCC016 cells were cultured in DMEM supplemented with 10% FBS and 1% PS. Both cell lines were maintained in a 37 °C humidified incubator containing 5% CO_2._

### 2.2. Development of Drug Resistant Cell Lines

Both HCC4006 and ETCC016 cell lines were treated with 5 μM erlotinib for over 10 passages to gain resistance to the drug. We named the resistant phenotype of these cell lines as HCC4006R and ETCC016R, respectively, whereas the parental control groups (called mock cell lines, i.e., HCC4006M and ETCC016M) were treated with the same amount of dimethyl sulfoxide (DMSO), instead of erlotinib.

### 2.3. MTS Assay

To evaluate the IC50 value of erlotinib in both drug-sensitive and drug-resistant cell lines, MTS assay CellTiter 96 Aqueous One Solution Cell Proliferation Assay kit (Promega, Madison, WI, USA) was performed. Briefly, 0.0001 µM, 0.001 µM, 0.01 µM, 0.1 µM, 1 µM, 10 µM, 20 µM, 50 µM, 100 µM, 200 µM erlotinib and control (DMSO) were added to both cell lines. After 72 h, the supernatant was discarded and replaced with MTS reagent solution (20 µL/well for 96-wellplate), and cells were then incubated for another 4 h. The absorbance of 490 nm wavelength was read by a plate-reader to measure the viability of cells.

### 2.4. Inducing Cell EMT

EMT in both HCC4006 and ETCC016 cell lines was activated through TGF-β receptor signaling pathways. Specifically, 4 ng/mL TGF-beta was added to the growth medium 36 h before experiment [11]. The occurrence/progression of EMT was monitored by examining the mRNA expression levels of N-cadherin, E-cadherin and vimentin in the cells.

### 2.5. Membrane Puncturing

Cells were digested and transferred into glass-bottom dish (MatTek, Ashland, MA, USA) precoated with poly-L-lysine (Sigma, St Louis, MO, USA), tested after 30 min incubation to ensure cells would adhere strongly to the dish. Membrane puncturing experiment was conducted using atomic force microscopy (AFM). Specifically, cylindrical AFM probes (with a diameter of 2 µm) were fabricated by focused ion beam (FEI Quanta 200 3D). After that, said probe was pushed into the cell to induce membrane damage (similar to that achieved by using laser or electric pulses [18]). The resealing of the created membrane pore was recorded with a phase contrast microscope.

### 2.6. Measuring Membrane Tension

The membrane tension of cells was estimated by indenting the cell membrane with a cylindric AFM, a method that has been used in previous studies [19,20]. Before contact, the intracellular (*P_c_*) and external (*P_e_*) pressures were balanced by the membrane tension *γ m* via the Laplace’s law as

(1) $$P_{c}-P_{e}=\frac{2\gamma_{m}}{R}$$

where *R* represents the cell radius. Since the indentation depth is small, the contact area A and indentation depth δ can be related to each other as:

(2) $$A=\pi \delta \;\left(2R-\delta \;\right)$$

On the other hand, the force *F* applied on membrane through the cross-section area A of the indenter must equal to that induced by the pressure difference across the membrane, i.e.,

(3) $$P_{c}-P_{e}=\frac{F}{A}$$

Combining the above equations, the membrane tension can be estimated as:

(4) $$\gamma_{m}=\frac{FR}{2\pi \delta \;\left(2R-\delta \;\right)}$$

### 2.7. Immunofluorescent Staining

Cells were treated with cold methanol for 10 min to make their membrane permeable to dyes (without destroying embedded membrane proteins), followed by another 10 min treatment of PBS with 0.25% Tween 20. After being washed with PBS for 3 times, the samples were immersed in 5% BSA blocking solution for incubating 30 min at room temperature. Next, primary monoclonal antibody annexin A2 (Thermo Fisher Scientific, Waltham, MA, USA) diluted in 2% BSA PBS and added. After overnight incubation at 4 °C and washing with PBS 3 times, a secondary antibody was added and the samples were incubated at room temperature for 1 hr. DAPI (4′,6-diamidino-2-phenylindole, Thermo Fisher Scientific, Waltham, MA, USA) was added into the medium 30 min before observation.

The fluorescence images were obtained using Nikon Eclipse Ts2R-FL fluorescence microscope with fixed exposure time for each sample.

### 2.8. Real Time Quantitative Polymerase Chain Reaction (RT-qPCR)

To evaluate the expression of EMT markers, real-time quantitative PCR (RT-qPCR) was employed to detect the expression levels of E-cadherin, N-cadherin and vimentin. To further determine the role of proteins in relation to membrane repairing capability, annexin A2, A5, A6, A7 were also tested. GAPDH was determined as the reference gene. The primers were purchased from Lifeact Technology (Thermo Fisher Scientific Waltham, MA, USA). TRIzol Reagent (Thermo Fisher Scientific, Waltham, MA, USA) was used to lyse the cell to extract mRNA, following the manufacturer’s instructions. RT-qPCR was performed with a QuantiNova SYBR Green PCR Kit (QIAgen, Hilden, Germany). Specifically, the cycling program was adopted as: (i) denaturation at 95 °C for 15 s, (ii) annealing at 60 °C for 30 s, and (iii) repeating these two steps for 45 cycles (Table 1).

## 3. Results

### 3.1. Fast Membrane Resealing Response Observed in NSCLC Cancer Cell Lines

**Figure 1:**
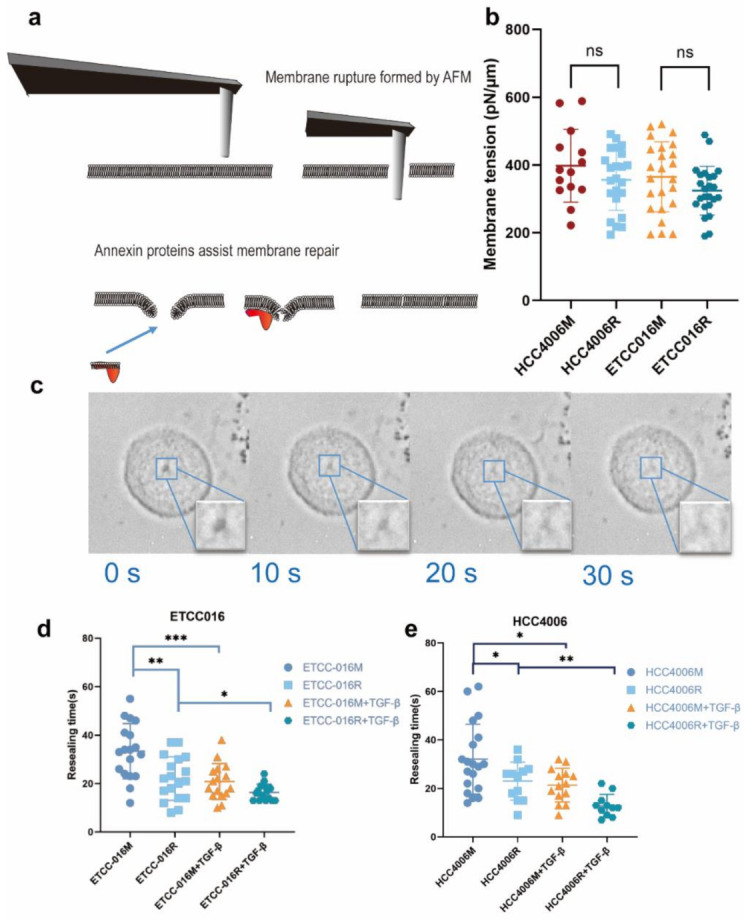
(**a**) An illustration of mechanical puncturing of the plasma membrane by a cylindrical AFM probe and its subsequent annexin-assisted self-repairing. (**b**) Membrane tension of different cells measured by AFM indentation. (**c**) A representative set of images showing the resealing of a membrane pore in the NSCLC cell line. Here, 0 s indicates the removal of the AFM probe. Clearly, the pore got smaller and smaller and eventually disappeared in ~30 s. (**d**,**e**) show the membrane resealing time measured from ETCC016 and HCC4006 cell lines under different treatments. Error bar represents standard deviation. (ns, not significant. ‘*’, ‘**’, ‘***’ represent *p* < 0.05, 0.01, 0.001, respectively, from unpaired *t*-test).

Using an atomic force microscope (AFM) mounted with a 2 μm cylindrical probe (refer to Figure 1a), membrane puncturing tests were conducted on two types of NSCLC cell lines, ETCC016 and HCC4006. Specifically, after retracting the AFM probe from the penetrated cell, self-repairing of the damaged membrane was monitored with the optical microscope. Interestingly, as shown in Figure 1c, most induced membrane pores can fully reseal within a few tens of seconds. Many factors have been reported to influence the membrane resealing response of cells. For example, physically, membrane tension acts to enlarge the pore. Therefore, the resealing time has been shown to increase exponentially with the membrane tension. To see whether this is important in the present study, we measured the membrane tension of different cell types used here via AFM indentation [19]. Interestingly, as illustrated in Figure 1b, no significant difference in the tension level among various cell types was observed, confirming that we do not need to consider the influence of membrane tension when analyzing different observed resealing responses of cells.

### 3.2. Membrane Resealing Speed Correlates with Cancer Cells’ Drug Resistivity

**Figure 2:**
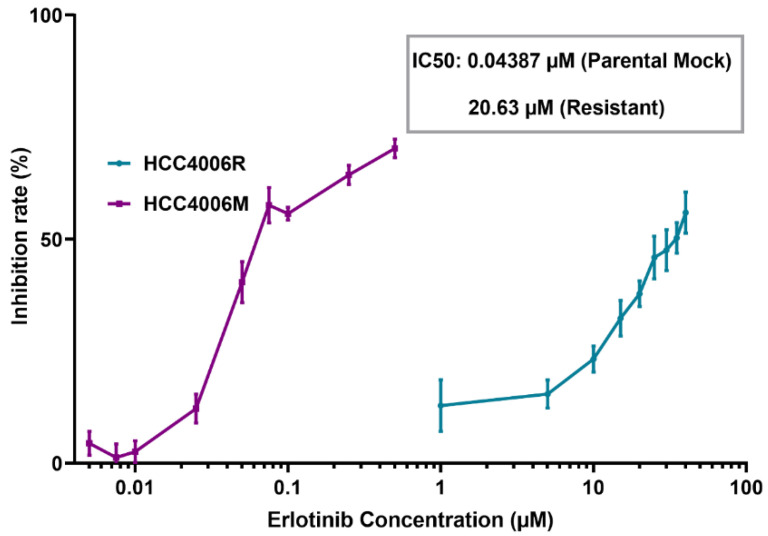
MTS assay of HCC4006 and HCC4006R cell lines. The drug resistance (HCC4006R) cell line was established by 10 cycles of drug treatment. Compared to drug-sensitive HCC4006M cells, a three orders of magnitude increase in the concentration of erlotinib is required to reduce the viability of HCC4006R cell line to 50%.

With the method for introducing membrane pores with precisely controlled size at hand, we can then examine how the self-repairing capability of cancer cells is influenced by their pathological status. Given its important clinical implications, we first investigated how the drug resistance developed in non-small cell lung cancer cells affect their membrane resealing ability. Specifically, drug-resistant ETCC016 and HCC4006 cells were developed by culturing these two cell lines with 5 μM erlotinib for more than 10 passages. The resistance to erlotinib of these cell lines was evaluated/verified by the MTS assay shown in Figure 2. Interestingly, as shown in Figure 1d,e, the resealing speed in drug-resistant (ETCC016R and HCC4006R) cells was found to be significantly faster than their drug-sensitive (ETCC016M and HCC4006M) counterparts. Specifically, on average, the 2 µm membrane pore in ETCC016R and HCC4006R cell lines could reseal in ~23 s whereas it took ~33 s for the holes in ETCC016M and HCC4006M cells to disappear.

**Figure 3:**
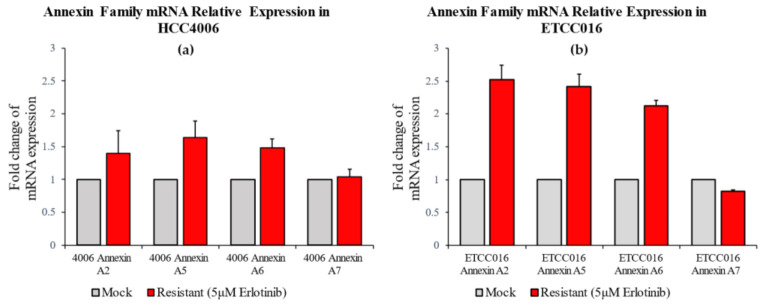
RT-PCR result of annexin A2, A5, A6, A7 expression levels in HCC4006 and ETCC016 cells, both for the mock (normal) and drug-resistant cell lines. Annexin A2, A5 and A6 were significantly more expressed in the resistant HCC4006 and ETCC016 (>2 folds) cell lines.

As pointed out earlier, membrane tensions in these drug-resistant and drug-sensitive cells were found to be more or less the same, therefore we look for other factors that could give arise to the different membrane resealing response observed here. In particular, we examined the expression of annexin in these cell lines, given the well-known role of such proteins in facilitating membrane remodeling. As shown in Figure 3a, for HCC4006 cell line, the expression levels of annexin A7 was found to be similar in both the mock and drug-resistant cells. However, the concentration of annexin A2, A5 and A6 are much higher in the drug-resistant cell line. Similarly, a 2-fold increase in the expression levels of annexin A2, A5 and A6 were also observed in ETCC016R cells compared to the control ETCC016M cell line (Figure 3b). Taken together, these evidences indicate that the enhanced membrane repairing capability in drug-resistant NSCLC cells was likely due to the overexpression of annexin (especially annexin A2, A5 and A6) in them. These four types of annexins were selected in the present study because annexin A2 can facilitate actin assembly near the membrane and consequently orchestrate its organization [21], annexin A5 is the most abundant membrane-bound annexin scaffold [22] while annexin A6 and A7 are known to be involved in the lipid-vesicle transport in cells [21,23].

### 3.3. Epithelial-Mesenchymal-Transition Leads to Faster Membrane Resealing of Cancer Cells

Another way for tumor cells to acquire drug-resistance is the EMT, where epithelial-like cells lose their apical-basal polarity and adhesion with each other, and eventually become highly migratory. Specifically, the reduced drug-induced apoptosis and elevated drug efflux pumping [24,25] in tumor cells undergoing such transition are believed to be behind their enhanced drug resistance. Given the correlation between the membrane resealing response and drug resistivity of cancer cells discussed above, it is interesting to see whether the membrane repairing capability of NSCLC cells is also regulated by EMT or not.

**Figure 4:**
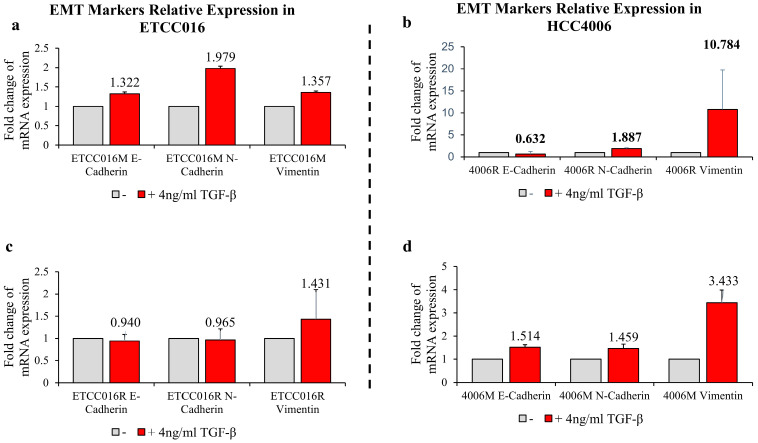
RT-qPCR results of EMT markers: E-cadherin, N-cadherin and vimentin. RT-qPCR experiments were performed after 36 h exposure with/without 4 ng/mL TGF–β. (**a**,**c**) Mock and resistant groups of ETCC016 cell line were induced EMT. (**b**,**d**) Resistant and mock groups of HCC4006 cell lines represent greater increase in vimentin expression, 10-fold increase in the resistant group and 3-fold increase in the mock group.

We proceeded by adding 4 ng/mL TGF-β, a common EMT inducer, into the culture medium of cells. Interestingly, as shown in Figure 1(d,e), the resealing of membrane pores in both HCC4006 and ETCC016 cell lines became faster after TGF-β treatment, confirming an enhanced self-repairing capability has been triggered. To confirm whether EMT was indeed induced by TGF-β treatment, the expression levels major EMT markers, e.g., vimentin and N-cadherin were measured by RT-qPCR. As illustrated in Figure 4, a 10-fold increase in the vimentin expression level in HCC4006R was observed, in which is far greater in the fold change compare to the drug-sensitive cell line (HCC4006M). For ETCC016 cells after TGF-β treatment, we observed that vimentin expressions were increased in both the resistant and mock cell lines.

**Figure 5:**
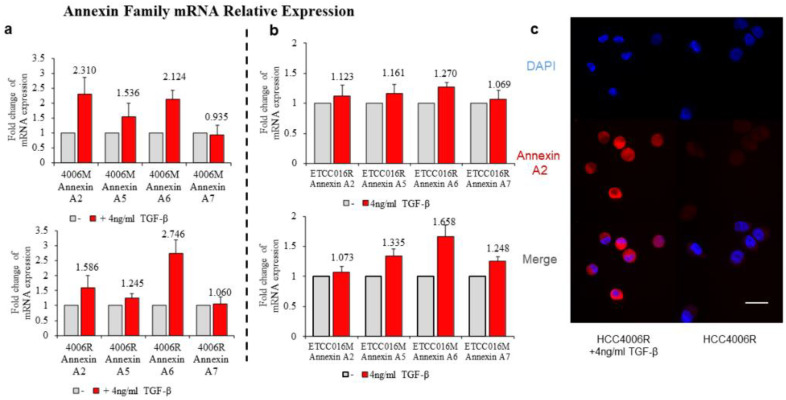
Annexin A2, A5, A6, A7 expression of HCC4006 (**a**) and ETCC016 (**b**) drug-resistant (R) and non-resistant (mock, M) cell line, with/without inducing EMT. (**c**) Immunofluorescence staining of annexin A2 protein for drug resistant HCC4006 cell lines with/without inducing EMT.

It must be pointed out that, among all cell lines tested, HCC4006R underwent the largest increase in the annexin expression level (after TGF-β treatment) coupled with the biggest decrease of membrane resealing time (from ~23 to ~13 s) which again supports the notation that EMT enhances the membrane repairing capability of cells. Similar to the previous drug-sensitive and drug-resistant case, we then examined how TGF-β treatment influences annexin expression in NSCLC cells. As expected, a much bigger increase in the expression level of annexin A2, A5, A6 and A7 was observed in drug-resistant HCC4006R cells, in comparison to the HCC4006M cell line (Figure 5a). On the other hand, such increase was fond to be more prominent in drug-sensitive ETCC016M cells compared to that in the ETCC016R cell line, see Figure 5b. These RT-qPCR results are consistent with our immunofluorescence staining observation where a significant increased annexin A2 fluorescent signal was detected in HCC4006R cells after TGF-β treatment, refer to Figure 5c.

## 4. Discussion and Conclusions

This study showed that micron-sized membrane pores (induced by AFM puncturing) in NSCLC cells could fully reseal in tens of seconds. Interestingly, although membrane tension remained more or less the same across cancer cell lines, erlotinib-resistant tumor cells were found to reseal their membranes much faster than the corresponding drug-sensitive cell lines. We further demonstrated that such enhanced membrane repairing capability was due to the overexpression of annexin (a protein family known to play significant roles in the remodeling and reorganization of lipid membranes) in drug-resistant NSCLC cells. Interestingly, EMT could further reduce the resealing time of the membrane. These findings are consistent with previous studies reporting that annexin facilitates membrane repairing by inducing curvature and folding of lipid bilayer near the pore edge [9], and knockdown of annexin A6 in cells will lead to impaired membrane healing [6].

It must be pointed out that clinically, overexpression of annexin A2 in the tumor was considered a predictor of adverse outcomes for patients [26]. Recent studies have also indicated that annexin is heavily involved in the development of cisplatin resistance in NSCLC [27,28]. Our study provides new insights into how annexin helps tumor cells improve their self-repairing ability, which is crucial for their survival in a hostile environment (caused by anti-cancer drugs or other disruptive factors). We would like to emphasize that the micron-sized nature of membrane pores introduced in the present study allows us to quantitatively monitor their resealing dynamics and unambiguously assess the membrane repairing capability of cells. Actually, we could also generate nano-sized membranes holes via techniques such as electroporation. However, it will be impossible to directly observe and measure the size evolution of those small pores. On the other hand, the biophysical mechanism behind the resealing of micro- and nano-sized holes should be similar. Therefore, the conclusions obtained here are expected to be robust.

Finally, given that membrane puncturing and resealing tests can be conducted in a few minutes, it is conceivable that we might be able to use membrane resealing time as a marker for rapid drug-resistance identification and/or classification of cancer. Indeed, cell stiffness level has been successfully applied to distinguish tumor cells from their normal counterparts [29]. Finally, we want to emphasize that, besides membrane tension and annexin considered here, factors such as the viscoelastic deformation [30,31] and dynamic remodeling [32,33] of the actin cortex could play a role in how membrane pores evolve as well. In addition, the cellular volume may also change (due to water efflux or influx [34,35]) during mechanical puncturing of the membrane, which then alters its tension level and eventually its resealing. More careful theoretical and experimental efforts are needed in the future to take these important features into account.

## Figures and Tables

**Table 1:** The sequences of primers for qPCR.

Gene Name	Forward Primer (5′-3′)	Reverse Primer (5′-3′)
E-Cadherin	TGCTGATGCCCCCAATACCCCA	GTGATTTCCTGGCCCACGCCAA
N-Cadherin	TGACTCCAACGGGGACTGCACA	AGCTCAAGGACCCAGCAGTGGA
Vimentin	AACCAACGACAAAGCCCGCGTC	TTCCGGTTGGCAGCCTCAGAGA
Annexin A2	TCGGACACATCTGGTGACTTCC	CCTCTTCACTCCAGCGTCATAG
Annexin A5	GTGGCTCTGATGAAACCCTCTC	GGCTCTCAGTTCTTCAGGTGTC
Annexin A6	GACTGACGAAGACACAATCATCG	CAGAATCAGCCTTGCCAGGTCT
Annexin A7	CGGATTGTGGTCACTCGAAGTG	CGGTAATCTCCACTCGTGTCAC
GAPDH	GTCTCCTCTGACTTCAACAGCG	ACCACCCTGTTGCTGTAGCCAA
